# Nrf2 Activation With CDDO-Methyl Promotes Beneficial and Deleterious Clinical Effects in Transgenic Mice With Sickle Cell Anemia

**DOI:** 10.3389/fphar.2022.880834

**Published:** 2022-05-10

**Authors:** Chibueze A. Ihunnah, Samit Ghosh, Scott Hahn, Adam C. Straub, Solomon F. Ofori-Acquah

**Affiliations:** ^1^ Department of Medicine, Center for Translational and International Hematology, Vascular Medicine Institute, School of Medicine University of Pittsburgh, Pittsburgh, PA, United States; ^2^ Heart, Lung, Blood and Vascular Medicine Institute, University of Pittsburgh, Pittsburgh, PA, United States; ^3^ Division of Hematology/Oncology, University of Pittsburgh, Pittsburgh, PA, United States; ^4^ School of Biomedical and Allied Health Sciences, University of Ghana, Accra, Ghana

**Keywords:** sickle cell anemia, nuclear factor erythroid 2-like 2 (Nrf2), CDDO-methyl (CDDO-Me), dimethyl fumarate (DMF), endothelin receptor (ET)

## Abstract

Activation of Nrf2, a major transcription factor that drives the antioxidant defense system, is an emerging therapeutic strategy in Sickle Cell Disease (SCD). In this study, transgenic Sickle Cell Anemia mice (SS mice) treated with CDDO-Methyl (CDDO-Me), a potent Nrf2 activator, showed reduced progression of hemolytic anemia with aging, but surprisingly also showed reduced endothelial function. Pulmonary vessels isolated from SS mice treated for 4 months with CDDO-Me displayed a diminished response to nitric oxide (NO)-induced vasodilation compared to littermates given vehicle. It is unclear what molecular mechanism underly the vascular impairment, however, our *in vitro* assays revealed that CDDO-Me induced the expression of the endothelin receptor (ET_A_ and ET_B_) in vascular smooth muscle cells. Endothelin signaling is associated with increased vascular tone and vasoconstriction. This study underscores the importance of pre-clinical benefit-risk investigations of Nrf2 activating compounds which may be used to treat patients with SCD.

## Introduction

Activation of the nuclear factor, erythroid 2-like 2 (Nrf2), a major transcription factor of the antioxidant defense system, is an emerging therapeutic strategy in Sickle Cell Disease (SCD). Nrf2 is a ubiquitously expressed basic leucine zipper (b-Zip) transcription factor. When stimulated by toxic insult, such as oxidative stress and inflammation, it facilitates the induction of approximately 200 antioxidant response (ARE) genes including glutathione-disulfide reductase (GSR), thioredoxin reductase-1 (TXNRD1) and heme oxygenase-1 (HO-1) (Thimmulappa et al., 2002; Lee et al., 2005).

The pathophysiology of SCD includes chronic intravascular hemolysis and anemia, leading to vasculopathy and endothelial dysfunction (Owusu-Ansah et al., 2015). Chronic and excessive vascular hemolysis releases cell-free plasma hemoglobin and arginase 1, which amplify the formation of reactive oxygen species, such as hydroxyl radicals. Cell-free hemoglobin scavenges nitric oxide (NO) through the deoxygenation reaction, enhancing the release of nitrate radicals. Collectively, these reactions intensify the vascular free radical burden, causing endothelial dysfunction and pathologic vascular remodeling (Gladwin et al., 2012). Further, clinical complications from SCD increase with aging, causing accelerated vascular and tissue damage, increased pain crises, and a median life expectancy that lags the general population (McKerrell et al., 2004; Thein et al., 2017).

Emerging clinical and animal data support the concept that activation of Nrf2 can induce antioxidant enzyme expression that could attenuate the enhanced intravascular oxidant load generated by chronic intravascular hemolysis in patients with SCD. A phase-1 open label dose-escalation study with the Nrf2 activator sulforaphane (SFN), increased the mRNA expression of heme oxygenase (HO-1) and NAD(P)H: Quinone Oxidoreductase (NQO1) in whole blood from patients taking SFN for 21 days (Doss et al., 2016). Another Nrf2 activator, CDDO-Imidazolide was found to be effective in reducing organ damage and relieving inflammation in sickle mice (Keleku-Lukwete et al., 2015).

In our previous publication, we reported the characterization of the age-related progression of SCD in transgenic Sickle Cell Anemia mice (SS mice), known as the adolescent-to-adult transition (Ghosh et al., 2016). We note that the age-related increase in intravascular hemolysis, organ damage, endothelial dysfunction, and cardiovascular sequalae observed in humans with SCD is analogous to the age-related complications observed in SS mice. Therefore, we used SS mice to show that prophylactic pharmacologic activation of Nrf2 with 3H-1,2 Dithiole-3-thione (D3T) during the adolescent-to-adult transition was efficacious to stabilize hemoglobin loss, ameliorate lung injury and reverse vascular inflammation with aging in SCD (Ghosh et al., 2016). We also reported that loss of Nrf2 in non-hematopoietic tissue was found to impact disease progression in non-hematopoietic tissues such as the vasculature, leading to endothelial dysfunction with aging in SS mice; most notably, loss of Nrf2 in non-hematopoietic tissue also worsened intravascular hemolysis with aging (Ghosh et al., 2016). Our results demonstrate the protective role which Nrf2 plays in non-hematopoietic tissue in the context of SCD. Further, we show the importance of Nrf2 activity in endothelial cells for the maintenance of healthy vascular tissue and healthy erythrocytes. Based on the observation that loss Nrf2 in non-hematopoietic tissue was found to worsen intravascular hemolysis and enhance endothelial dysfunction with aging, we hypothesized that a drug which activates Nrf2 in vascular endothelial cells, would improve endothelial dysfunction with aging and protect HbS erythrocytes in a paracrine manner. In this paper, we tested our hypothesis in a cohort of SS mice.

## Materials and Methods

### Nanostring Array

We used nCounter technology from Nanostring to profile the induction of 104 cytoprotective, antioxidant, and heme metabolizing Nrf2 target genes after 12 h of drug treatment. Human microvascular endothelial cells (HMVECs) and human erythroleukemia cells (K562) were cultured for 12 h with DMF 20 μM (Sigma), CDDO-Me 100 nM (Sigma) or vehicle (DMSO). RNA was isolated using RNeasy mini kits (Quiagen). 500 ng of RNA was analyzed using the nCounter analysis system through the University of Pittsburgh Genomics Core. Fold induction was calculated using the nSolver software from Nanostring. For nSolver software analysis, raw values were normalized to 10 house-keeping genes including (ACTB, B2M, GAPDH, GUSB, HMBS, HPRT1, RPL13A,SDHA,TBP,UBC,YWHAZ). Fold induction was calculated as a ratio of normalized drug treated samples versus normalized DMSO treated samples. Two-tailed *t*-tests were performed on log transformed normalized data that assumes unequal variances, *p* < 0.05 was considered significant. False discovery rate was calculated using the Benjamini-Yekutielli procedure, the chosen cutoff value was 0.2. For HMVEC and K562 cells, fold induction cutoff ≥1.5, FDR cutoff ≤0.2, *n* = 3-6, biological replicates, *p* < 0.05.

### Cell Culture

Primary human lung microvascular endothelial cells (HMVECs, Lonza#CC2527) were cultured using endothelial cell growth medium (Lonza). Human erythroleukemia cells (K562, American Type Culture Collection) were cultured in Iscove’s Modified Dulbecco Medium (ThermoFisher Scientific), rat and human smooth muscle cells (American Type Culture Collection) were cultured in Smooth Muscle Growth Medium (Lonza). For pharmacological experiments cells were culture in DMF (10–40 μM), CDDO-Me (50–200 nM) or DMSO for 6–48 h. All cells were cultured in a cell culture incubator (Applied Biosystems) under standard conditions at 37°C and 5% CO_2._

### Western Blot

Nuclear and cytoplasmic extracts were prepared using NE-PER kits (ThermoFisher Scientific). Whole cell lysates were prepared using IP lysis buffer (ThermoFisher Scientific. Immunoblots were performed using standard electrophoresis and western blotting chamber (Bio-Rad) with 20–40 μg of total protein lysate. Antibodies used were Nrf2 (Abcam, #ab31163) and mutS homolog 2 (MSH2) (Abcam, # ab70270), Quantification of western blots was done using NIH Image J processing with triplicate biological replicates according to the recommended procedures described by the NIH. In short, the values represent the mean pixel density for all data. For all western bands the same area of analysis was used to analyze each band. The bar graphs represent the values that were calculated according to the following formula, (band/control + background).

### Animals Procedures

Male and female Townes Sickle Cell Anemia (SS) mice were used, and mouse genotypes were confirmed by PCR (Wu et al., 2006). For treatment with CDDO-Methyl Ester (Sigma,# SMB00376) 1-month old sickle mice received CDDO-Me (20 μmol/kg BW) or DMSO three times per week by oral gavage for 4-month. Mice were phlebotomized under anesthesia by retro-orbital bleeding using a capillary tube internally coated with heparin/EDTA anticoagulant. Animal to human dose equivalent calculations were based upon body surface area normalization published used by the US Food and Drug Administration and published in the publicly available *Guidance for Industry, Estimating the Maximum Safe Starting Dose in Initial Clinical Trials for Therapeutics in Adult Healthy Volunteers* (2005).

### Dosing Selection for Cell Culture

The doses chosen were based upon the results from the preliminary dose-response analysis of the mRNA induction of the canonical target genes of interest HO-1, NQO1, GCLM, and GCLC (data not shown); note we also considered the average plasma concentration found in patients prescribed each drug or in clinical trials (Linker and Gold, 2013; Wang et al., 2014). Our preliminary experiments (data not shown) revealed that CDDO-Me stimulated the most robust response of the canonical target genes of interest at nanomolar concentrations (50–100 nM), while DMF elicited comparable but lower induction at micromolar concentrations (10–20 μM).

### Drug Formulation for Animal Treatment

CDDO-Me was initially solubilized in DMSO to prepare concentrated stock aliquots. The CDDO-Me (20 μmol/kg/TIW) or Vehicle (DMSO) formulation administered to SS mice by oral gavage was prepared using 25% Glycerol (Sigma, #G5516) and 1% Kalliphor-EL (Sigma, #C5135) in 1X PBS brought to a final volume of 50–100 μL per mouse.

### Plasma Analysis

Freshly collected EDTA anticoagulated blood samples were centrifuged at 1,200 × g for 15 min to collect plasma. sVCAM was quantified using quantikine ELISA kits from R&D Systems (#MVC00) following manufacturer’s instructions.

### Reticulocyte Percentage/Complete Blood Count

Reticulocyte percentage was measured in venous whole blood using a HemaTrue hematology analyzer (Heska). To assess drug induced changes in total reticulocyte percentage we compared baseline reticulocyte values to values from the same mouse treated with CDDO-Me or vehicle after 16 weeks.

### Whole Blood Co-Oximetry

Total hemoglobin (Hb) concentration was measured in venous whole blood using a portable CO-oximeter (AVOXImeter 4000, ITC) as previously described (Ghosh et al., 2016). To assess drug induced changes in total Hb concentration we compare baseline Hb values to Hb values from the same mouse treated with CDDO-Me or vehicle after 16 weeks.

### Tissue Myography

Lungs were rapidly excised and placed in ice-cold physiological salt solution (PSS) containing (in mM): NaCl 118, KCl 4.7, MgSO_4_ 1.17, KH2 PO4 1.18, D-glucose 6, NaHCO_3_ 4, Hepes 10, pH 7.4. CaCl_2_ (2.5 mM) was added prior to experiment. Pulmonary arteries were dissected from the left and right lung lobes and cut into 2 mm rings. 40 μm wires were threaded through the lumen of the ring and placed onto a wire myograph (DMT 620M). Following 30 min incubation vessels were stretched to 13.3 kPa, corresponding to a transmural pressure of 100 mmHg. Viability was tested by 45 mM KCl exposure for 15 min. Rings were washed 3 times with PSS and allowed to rest for 30 min.

After the initial test of viability, arterial segments were contracted with a continuous dose response of endothelin-1 (100 pM-10 nM). After reaching plateau, endothelial function was examined with a continuous dose response curve of acetylcholine (10 nM-100 mM) to produce relaxation. 80 mM KCl was added to again check viability and was followed by Ca^+^ free PSS containing 100 μM sodium nitroprusside for maximal dilation.

### Real-Time PCR

RNA was isolated using RNeasy mini kits (Quiagen) according to the manufacturers’ protocol. Nanodrop quantification was performed, and 500 ng of total RNA was converted to cDNA using high-capacity reverse transcription kits (Applied Biosystems). Gene expression analysis was performed using the Stepone Plus thermal cycler (Applied Biosystems) and Taqman probes for each gene of interest and housekeeping genes (Invitrogen). Relative quantification was calculated using the standard ΔΔCT method. For human cell experiments (HMVEC, K562, human smooth muscle cell), target gene transcripts were normalized to the 18S ribosomal transcripts. For rat cell experiments (rat smooth muscle cells), target gene transcripts were normalized to beta actin transcripts. Taqman probes used: human ribosomal 18 s assay Hs99999901_s1, human gamma globin assay Hs00361131_g1, human endothelin receptor alpha assay Hs03988672_m1, human endothelin receptor beta assay Hs00240747_m1, human beta actin assay Hs01060665_g1, rat endothelin receptor alpha assay Rn00561137_m1, rat endothelin receptor beta assay Rn00569144_m1, rat beta actin assay Rn00667869_m1.

### Statistical Analyses

Results are reported as mean ± SEM or SD. To analyze statistical significance, two-tailed paired or unpaired Student’s t-test, two-way analysis of variance (ANOVA) with Bonferroni post-tests, and repeat-measures two-way ANOVA were used as appropriate. *p*-values of less than 0.05 were considered significant. GraphPad Prism 6 software was used for all statistical analyses except for the Nanostring data which used nSolver software as described.

## Results

We designed a biological assay to screen potential drug candidates and determine the most efficacious candidate to test our hypothesis in SS mice. The biological assay was a multiplex mRNA screening array (nCounter) which could profile the induction of dozens of Nrf2 target genes simultaneously in each sample. Nrf2 is known to upregulate at least 200 different genes which modulate a wide array of biological processes (Thimmulappa et al., 2002; Lee et al., 2005). However, our interest was in the induction of cytoprotective, antioxidant, and heme metabolizing Nrf2 target genes. Note, the genes that we hypothesized would be the most clinically relevant for SCD patients are heme-oxygenase (HO-1), NAD(P)H quinone dehydrogenase 1 (NQO1), glutamate cysteine ligase modifier subunit (GCLM) and glutamate cysteine ligase catalytic subunit (GCLC). Those genes may offer therapeutic benefit for SCD patients because they are primarily involved in heme metabolism and/or cytoprotection. In addition to the aforementioned canonical target genes, our determination of the most efficacious candidate compound, was based upon which drug induced the *highest number* of Nrf2 target genes that are known to be associated with the antioxidant response in endothelial cells. To determine the most common 100 Nrf2 target genes associated with the antioxidant and cytoprotective response, we conducted a pathway analysis for genes of interest which relied upon published literature and publicly available biomedical databases (PubMed, EMBASE, Cochrane Library, TOXNET, Scopus, and Web of Science).

The test compounds we considered for the screening assay were chosen based upon their reported ability to activate Nrf2 and their previous or current use clinically. Further, we performed a dose-response analysis (data not shown) to assess which compounds induced the most robust mRNA induction of the canonical target genes of interest, including HO-1, NQO1, GCLM and GCLC. Ultimately, the Nrf2 activators dimethyl fumarate (DMF) and CDDO-Methyl (CDDO-Me) were chosen as potential candidates for the mRNA screening assay. Both drugs have shown clinical promise; DMF is an FDA approved drug marketed under the brand name Tecfidera, indicated for the treatment of multiple sclerosis (Linker and Haghikia, 2016), and CDDO-Me has been evaluated for efficacy in clinical trials with patients with moderate-to-severe chronic kidney disease (Pergola et al., 2011a; Hong et al., 2012). See the methods section for a description of the dosing selection for cell culture.

To identify which drug could induce the most cytoprotective genes in vascular tissue, we treated human microvascular endothelial cells (HMVEC cells) and K562 cells (human erythro-leukemia cell-line). Note, our primary interest was to determine which drug could induce the antioxidant response in endothelial cells, however, we considered a secondary interest in the induction of Nrf2 in erythroid progenitors’ cells because erythroid cells are directly affected in SCD. Further, we chose human endothelial and erythrocyte cells to maintain clinical relevance when we interpret the results from the *in vivo* study conducted in SS mice.

**FIGURE 1:**
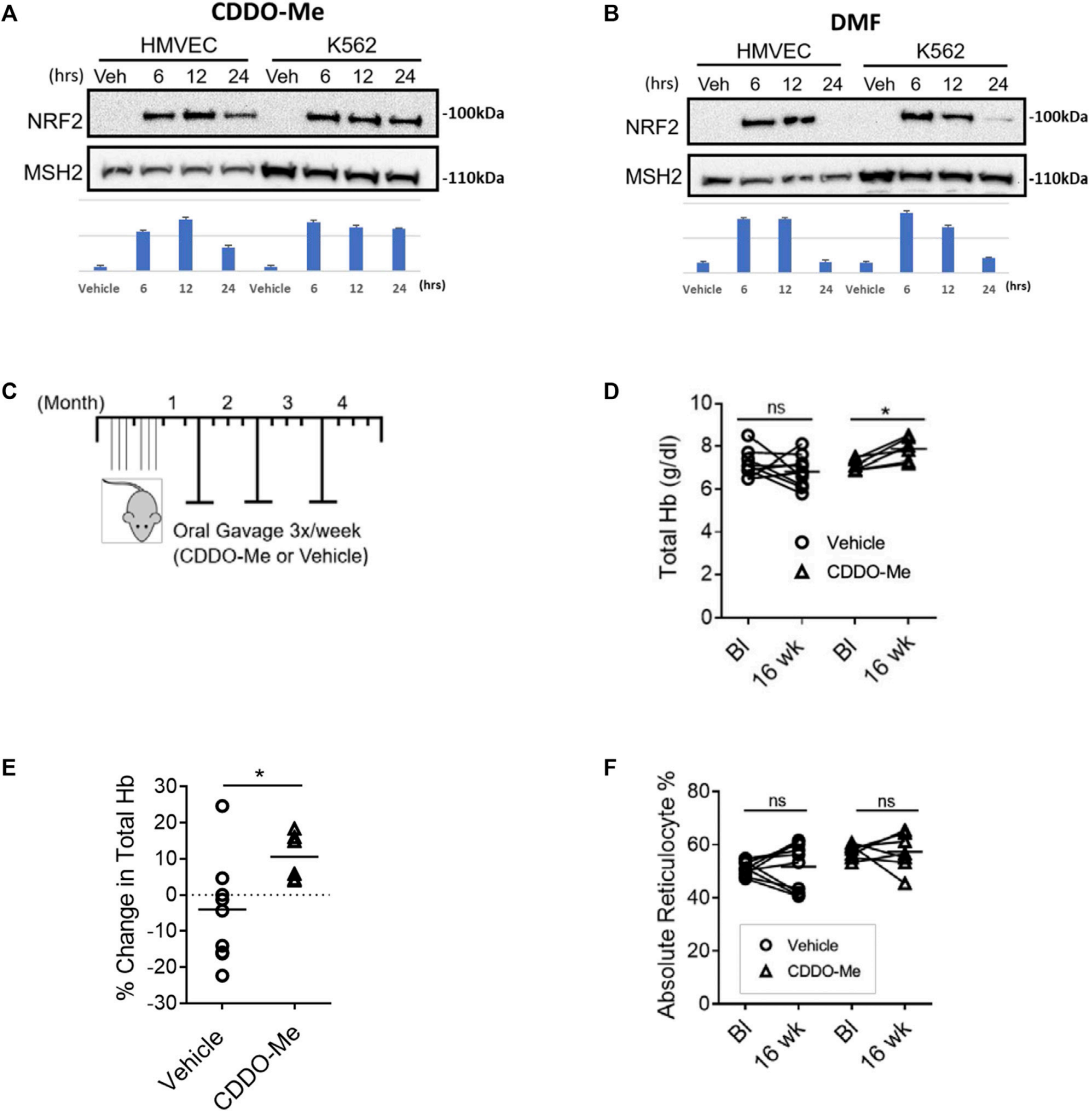
CDDO-Me is a potent activator of endothelial and erythroid NRF2 *in vitro* and improves hemolytic anemia with aging in SS mice. **(A,B)** Immunoblots were used to assess drug specific temporal differences in Nrf2 nuclear translocation in HMVEC and K562 cells. Quantification was done using NIH Image J processing with aribitrary units shown for the *y*-axis. Cells were cultured and treated with **(A)** CDDO-Me (100 nM) or vehicle, and **(B)** DMF (20 μM) or vehicle for 6, 12, and 24 h. Subsequently, nuclear extracts were isolated and probed for NRF2 and the loading control mutS homolog 2 (MSH2). **(C–F)** SS mice were randomly chosen at 4–6 weeks of age to be treated for 16 weeks with CDDO-Me (20 μmol/kg) or vehicle (DMSO) by oral gavage. **(C)** Schematic illustrating the experimental dosing regimen in SS mice. To assess drug induced changes in hematological chemistry with aging, mice were phlebotomized with retro-orbital bleeds to compare baseline (Bl) values to values from the same mouse after 16 weeks (16 weeks). For total hemoglobin quantification, we used a portable Co-Oximeter. Pairwise changes in **(D)** absolute total hemoglobin values and **(E)** total hemoglobin percent change are shown (*n* = 6 CDDO-Me group, *n* = 10 vehicle group, ^∗^ *p* < 0.05, Student’s t-test). **(F)** For reticulocyte quantification we used a Heska HemaTrue analyzer to compare baseline and 16-week values for SS mice treated with CDDO-Me (20 mmol/kg) or vehicle (DMSO) by oral gavage. Pairwise changes in absolute reticulocyte percentage values are shown (*n* = 6 CDDO-Me group, *n* = 10 vehicle group, ^∗^ *p* < 0.05, Student’s t-test).

HMVEC and K562 cells were cultured for 12 h with DMF (20 μM), CDDO-Me (100 nM) or vehicle (DMSO) followed by mRNA extraction, nCounter array evaluation, and analysis. Our experiment revealed that CDDO-Me induced more Nrf2 target genes than DMF in HMVEC (22 > 18) and K562 (14 > 11) cells (Tables 1, 2). Both drugs induced key heme metabolizing (HO1, FTH1, FTL) and cytoprotective (NQO1, G6PD, GSR, GCLM, GCLC) enzymes, however, CDDO-Me induced additional antioxidant enzymes in HMVEC, as well as gamma globin in K562 cells (Tables 1, 2). Gamma globin drives the expression of fetal hemoglobin (HbF), which is known to be therapeutic is SCD(Perumbeti et al., 2009). We used immunoblots to show drug-specific temporal effects driving the nuclear translocation of Nrf2, and subsequent gene expression in each cell type. CDDO-Me treatment caused sustained nuclear translocation of Nrf2 in both cell types, while Nrf2 nuclear translocation in DMF treated cells was more transient (Figures 1A,B). These results suggested that CDDO-Me may be an efficacious activator of the endothelial antioxidant defense, and concurrently may be cytoprotective in erythroid cells. Based on the results from the Nanostring array and our preliminary gene expression assessments, we chose CDDO-Me as the candidate for a longitudinal 16-week *in vivo* study in a cohort of transgenic sickle (SS) mice.

**TABLE 1:** Drug specific cytoprotective gene induction in HMVEC.

Drug	Gene name	Gene abbreviation
CDDO-Me	ATP binding cassette subfamily B member 11	ABCB11
	ATP binding cassette subfamily C member 2	ABCC2
	ATP binding cassette subfamily G member 2	ABCG2
	Aldo-keto reductase family 1 member B	AKR1B1
	Aldo-keto reductase family 1 member C2	AKR1C2
	Glutathione S-transferase alpha 4	GSTA4
	Microsomal glutathione S-transferase 1	MGST1
DMF	ATP binding cassette subfamily C member 1	ABCC1
	Aldo-keto reductase family 1 member B10	AKR1B10
	peroxiredoxin 1	PRDX1
Both	ATP binding cassette subfamily C member 3	ABCC3
	Aldo-keto reductase family 1 member C3	AKR1C3
	Carbonyl reductase 1	CBR1
	Epoxide hydrolase 1	EPHX1
	Ferritin heavy chain 1	FTH1
	Ferritin light chain	FTL
	Glucose-6-phosphate dehydrogenase	G6PD
	Glutamate-cysteine ligase catalytic subunit	GCLC
	Glutamate-cysteine ligase modifier subunit	GCLM
	Glutathione-disulfide reductase	GSR
	Heme oxygenase 1	HMOX1
	NAD(P)H quinone dehydrogenase 1	NQO1
	Sequestosome 1	SQSTM1
	Thioredoxin	TXN
	Thioredoxin reductase 1	TXNRD1

We used nCounter technology from Nanostring to profile the induction of 104 cytoprotective, antioxidant, and heme metabolizing Nrf2 target genes after 12 h of drug treatment in Human microvascular endothelial cells (**HMVECs**, Table 1) and human erythroleukemia cells (**K562**, Table 2). HMVEC’s and K562 cells were cultured for 12 h with DMF 20 μM (Sigma), CDDO-Me 100 nM (Sigma) or vehicle (DMSO). RNA was analyzed using the nCounter analysis system through the University of Pittsburgh Genomics Core. Fold induction cutoff ≥1.5, FDR cutoff ≤0.2, *n* = 3-6, biological replicates, *p* < 0.05.

**TABLE 2:** Drug specific cytoprotective gene induction in K562 cells.

Drug	Gene name	Gene abbreviation
CDDO-Me	Hemoglobin subunit gamma 1	HBG1
	MAF bZIP transcription factor G	MAFG
	Glutamate-cysteine ligase catalytic subunit	GCLC
	Heme oxygenase 1	HMOX1
DMF	Sequestosome 1	SQSTM1
Both	Aldo-keto reductase family 1 member C2	AKR1C2
	Aldo-keto reductase family 1 member C3	AKR1C3
	Epoxide hydrolase 1	EPHX1
	Ferritin heavy chain 1	FTH1
	Ferritin light chain	FTL
	Glutamate-cysteine ligase modifier subunit	GCLM
	Glucose-6-phosphate dehydrogenase	G6PD
	Glutathione-disulfide reductase	GSR
	NAD(P)H quinone dehydrogenase	NQO1
	Thioredoxin reductase 1	TXNRD1

We used nCounter technology from Nanostring to profile the induction of 104 cytoprotective, antioxidant, and heme metabolizing Nrf2 target genes after 12 h of drug treatment in Human microvascular endothelial cells (**HMVECs**, Table 1) and human erythroleukemia cells (**K562**, Table 2). HMVEC’s and K562 cells were cultured for 12 h with DMF 20 μM (Sigma), CDDO-Me 100 nM (Sigma) or vehicle (DMSO). RNA was analyzed using the nCounter analysis system through the University of Pittsburgh Genomics Core. Fold induction cutoff ≥1.5, FDR cutoff ≤0.2, *n* = 3-6, biological replicates, *p* < 0.05.

To test whether prophylactic CDDO-Me treatment could improve total hemoglobin loss during the adolescent-to-adult transition, we treated a cohort of 4-6-week-old SS mice with CDDO-Me (20 μmol/kg/TIW); dose equivalent to a human dose of 50 mg (0.8 mg/kg for a 60 kg adult) or vehicle for 16 weeks by oral gavage (Figure 1C). The dose chosen was based on the published doses provided to patients in clinical trials which range from 25–150 mg/day. We chose the equivalent of 50 mg/day because that dose appeared to be moderate and well tolerated in clinical trials (Pergola et al., 2011b). After 16 weeks of treatment, we reported several findings. Total hemoglobin (Total Hb) stabilized and increased by an average value of 10% in the CDDO-Me group, conversely, a 5% decrease in total Hb was noted in the vehicle-treated group (*p* < 0.05) (Figures 1D,E). Surprisingly, we did not observe any aggregate differences in reticulocyte number between the two groups (Figure 1F).

**FIGURE 2:**
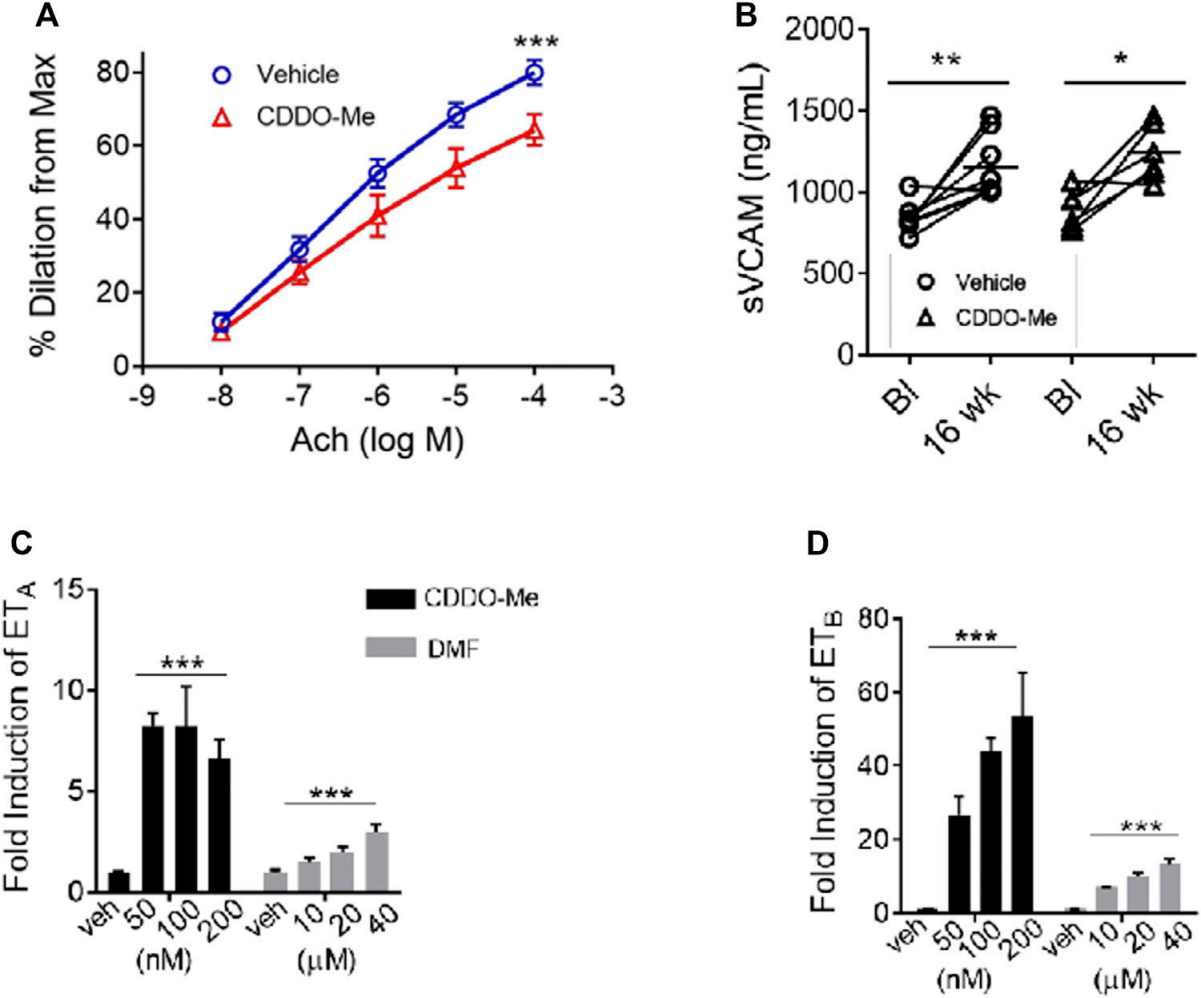
CDDO-Me worsens the vasodilatory response of pulmonary artery vessels from SS mice. CDDO-Me and DMF induced the genetic expression of the endothelin receptor A (ET_A_) and B (ET_B_) in rat smooth muscle cells. SS mice were randomly chosen at 4–6 weeks of age to be treated for 16 weeks with CDDO-Me (20 μmol/kg) or vehicle (DMSO) by oral gavage as described in Figure 1. **(A)** For nitric oxide (NO) response analysis, we used wire myography which quantified changes in vessel vasodilation and vasoconstriction. Lungs of SS mice treated for 16 weeks with CDDO-Me or vehicle were contracted with a continuous dose response of Endothelin-1 (100 pM-10 nM). After reaching plateau (max constriction), endothelial function was examined with a continuous dose response curve of acetylcholine (10 nM–100 mM) to produce relaxation (*n* = 5 for each group, ^∗∗∗^ *p* < 0.001, 2-way ANOVA).**(B)** We used ELISA assays for soluble vCAM to assess the effect CDDO-Me had on inflammation. Plasma samples were isolated from whole blood collected at baseline and after 16 weeks of treatment (vehicle *n* = 8, CDDO-Me *n* = 8; ^*^ *p* < 0.05, ^∗∗^ *p* < 0.01, ns, not significant, Student’s t-test). Real-time PCR was used to analyze and compare the drug-induced temporal expression of the endothelin receptor A (ET_A_) and B (ET_B_). **(C,D)** We cultured rat smooth muscle cells and treated them with CDDO-Me (50,100, and 200 nM), DMF (10, 20, and 40 μM) or vehicle for 6–24 h. RNA was isolated for cDNA synthesis and analyzed using real-time PCR (*n* = 6 biological replicates; ^∗^ *p* < 0.05, ^∗∗^ *p* < 0.01, ^∗∗∗^ *p* < 0.001, NS, not significant).

In SCD, inadequate endothelial nitric oxide synthase function (eNOS) can promote endothelial dysfunction through a complex process called eNOS uncoupling (Wood et al., 2006). Smooth muscle vasodilation can be measured as a surrogate for adequate eNOS function. To assess if CDDO-Me improved eNOS function, pulmonary artery vessels from SS mice treated with CDDO-Me (20 μmol/kg/TIW) or vehicle for 16 weeks were analyzed. We used tissue myography to pre-constrict pulmonary artery vessels with endothelin-1 until they reached plateau (max constriction), then acetylcholine (ACh) was used to induce NO release and subsequent smooth muscle vasodilation. Surprisingly, pulmonary arteries isolated from the CDDO-Me treated SS mice showed a reduction in vasodilation compared to the arteries isolated from vehicle treated littermates (vehicle *n* = 5, CDDO-Me *n* = 5; *p* < 0.001) (Figure 2A).

There was no indication that CDDO-Me induced local inflammation which could decrease NO production, as shown by the plasma soluble vCAM comparison (Figure 2B). Our results suggested that CDDO-Me may be facilitating the observed vascular response through another Nrf2 target gene that affects vascular tone. We decided to investigate whether CDDO-Me could regulate the expression of the endothelin receptor in the smooth muscle layer of the vessel wall.

To test that idea, rat smooth muscle cells (rSMC) were treated with CDDO-Me (50–200 nM) or vehicle. Our results showed significant upregulation of the endothelin receptor subtype A (ET_A_) mRNA and subtype B (ET_B_) mRNA (*p* < 0.01, *n* = 6) by CDDO-Me (Figures 2C,D). Interestingly, DMF treatment (10–40 μM) also induced endothelin receptor expression in the rSMC, (*p* < 0.01, *n* = 6) albeit with less potency than CDDO-Me at all concentrations tested (Figures 2C,D).

## Discussion and Conclusion

### Study Summary

The results of our study illustrate a novel benefit-risk profile for CDDO-Me when used to treat complications associated with the age-related progression of SCD. We hypothesized that an Nrf2 activator which induced genes associated with the antioxidant response in endothelial cells would attenuate endothelial dysfunction in SS mice with aging. Further, we presumed the increased antioxidant expression from vascular cells may protect HbS erythrocytes in a paracrine manner. We showed that during the adolescent-to-adult-progression, CDDO-Me can stabilize and improve hemolytic anemia in SS mice. However, CDDO-Me appears to have worsened endothelial dysfunction in treated SS mice. Surprisingly, our results showed that pulmonary artery vessels from SS mice treated with CDDO-Me were less sensitive to NO stimulated vasodilation than control SS mice. We suspect that this interesting finding may be due to Nrf2’s ability to modulate the endothelin pathway.

### Potential Role for Endothelin Signaling

The endothelin pathway consists of the endothelin peptide, and two endothelin receptor isoforms, type A (ET_A_) and B (ET_B_). Endothelin is a vasoactive peptide produced by endothelial cells, and the endothelin receptors are expressed in many tissues, including smooth muscle. Endothelin signaling is important for the regulation of vascular tone (Enevoldsen et al., 2020). Modulation of the endothelin pathway by Nrf2 may explain the vasoconstrictive effect we observed on pulmonary arteries from mice treated with CDDO-Me. Recently, it was reported by Kansanen et al., that nitro-oleic acid, a nitro-fatty acid, and activator of Nrf2, could induce the expression of the endothelin receptor B in mouse endothelial cells. Further, the authors’ reported that a putative antioxidant response element (ARE) was identified in the promotor region of the gene for both endothelin receptor subtypes (Kansanen et al., 2017). Those results support our finding that activation of Nrf2 with CDDO-Me and DMF can upregulate the expression of the endothelin receptor (ET_A_ and ET_B_) in rodent and human smooth muscle cells (Figures 2C,D). Note, we did not directly measure the mRNA expression of ET_A_ and ET_B_ in smooth muscles of treated SS mice, therefore, it is unclear if the upregulation of endothelin receptor isoforms A and B does, in fact, worsen endothelial dysfunction. However, pharmacologic antagonism of endothelin receptors has been shown to be an effective antihypertensive therapeutic strategy used clinically. Several endothelin receptor antagonists are approved by the FDA including bosentan and ambrisentan and are indicated for the treatment of pulmonary artery hypertension (PAH) (Steiner and Preston, 2008; Enevoldsen et al., 2020).

The induction of the endothelin receptor by CDDO-Me and DMF in rat and human cell types provides a potential mechanism which explains why pulmonary artery vessels from SS mice treated with CDDO-Me were less sensitive to NO stimulated vasodilation than control SS mice. While we acknowledge that follow up experiments are necessary to confirm that hypothesis, additional clinical evidence suggests that CDDO-Methyl modifies the endothelin signaling pathway leading to cardiovascular toxicities. In the BEACON study, where CDDO-Methyl was given to patients with chronic kidney disease and Type 2 Diabetes Mellitus, the study had to be prematurely terminated due to a high occurrence of cardiac events including high blood pressure and heart failure in CDDO-methyl treated patients. However, in the pre-clinical arm of that study, monkeys treated with CDDO-methyl for 28-day were found to have reduced protein expression of ET_A_ in the renal papilla and medulla. Further, rat proximal tubular cells treated with CDDO-methyl were shown to have lower expression of endothelin-1 after 24 h (Chin et al., 2014).

These results, when considered with our findings and the finding from other laboratories (Kansanen et al., 2017), suggest that CDDO-Me may have cell and species-specific effects regarding the modulation of endothelin signaling. Additional studies are certainly necessary to parse the spatial and species-specific pharmacodynamics of Nrf2 signaling on the endothelin pathway.

### Limitations and Future Studies

It is not clear if the *in vivo* adverse effects that we report are specific to CDDO-Me, or if they could potentially be replicated with other Nrf2 activators. Further, it is also unclear if the effects reported here are Nrf2 dependent or if they are off target effects that are Nrf2 independent. Dimethyl fumarate is approved by the US FDA for the treatment of multiple sclerosis (Bomprezzi, 2015). To date, the publicly available drug facts label does not carry a warning regarding drug induced cardiovascular events. Also, the potential to develop cardiovascular toxicity from taking dimethyl fumarate has not been reported in the medical literature. Note, it has been reported by To et al., that CDDO-Me and DMF have the ability to regulate different subsets of Nrf2 associated genes. Further, DMF and triterpenoids such as CDDO-Me have the ability to acitivate other pathways that are not connected to Nrf2, such as IkappaB (IKK) and PI3K/Akt. This is most likely due to the electrophilic Michael acceptor sites on those compounds which can interact with nucleophilic reactive cysteines on target proteins and elicit a signaling response (To et al., 2015). Additionally, considering that constitutive activation of Nrf2 did not appear to elicit any cardiovascular toxicities in a cohort of transgenic sickle cell anemia mice (Keleku-Lukwete et al., 2019), additional studies are needed to determine if the adverse effects reported here are drug specific or drug class specific.

One noteworthy finding which we report here, is the stabilizlaiton of total hemoglobin in the CDDO-Me group, despite no difference in absolute reticulocyte percentage between the treated and control group. In the future, an in-depth analysis of the hematology parameters will be necessary to investigate the cause out this finding. For example, an assessment of the mean corpuscle volume (MCV), mean corpuscular hemoglobin (MCH), mean corpuscular hemoglobin concentration (MCHC) and hematocrit (HCT) could provide useful information to determine what effect CDDO-Me may have on red blood cell stabilization. One potential explanation may be the anti-oxidant effect CDDO-Me might stimulate on the vessel wall. As described in the introduction, SCD patients suffering from chronic intravascular hemolyiss are subject to elevated levels of oxidative stress from the release of redox sensitive hemoglobin. Our hypothesis was that CDDO-Me could induce the transcription of anti-oxidant and cytoprotective enzymes such as NQO1 and HO-1 in the endothelial cells lining the vasculature. In turn, a healthy vessel wall may induce a paracrine response that is benficial to the local vascular envirionemnt, including red blood cells. In the future, it will be necessary to isolate endothelial cells from treated mice and control mice to investigate this potential explanation.

Nrf2 activation as a therapeutic strategy to treat SCD is a burgeoning concept but our results should provide a cautionary warning. Further studies are needed to verify the potential adverse effect that we report in this study, however, this study provides evidence of potential toxicological targets that should be considered when selecting Nrf2 activating drug candidates for patients with SCD.

## Data Availability

The datasets presented in this study can be found in online repositories. The names of the repository/repositories and accession number(s) can be found below: https://www.ncbi.nlm.nih.gov/geo/, GSE130432.
